# Tree xylem water isotope analysis by Isotope Ratio Mass Spectrometry and laser spectrometry: A dataset to explore tree response to drought

**DOI:** 10.1016/j.dib.2020.105349

**Published:** 2020-02-29

**Authors:** Simon Damien Carrière, Nicolas K. Martin-StPaul, Coffi Belmys Cakpo, Nicolas Patris, Marina Gillon, Konstantinos Chalikakis, Claude Doussan, Albert Olioso, Milanka Babic, Arnaud Jouineau, Guillaume Simioni, Hendrik Davi

**Affiliations:** a INRAE, UMR 1114 EMMAH, Domaine Saint Paul, INRAE Centre de Recherche PACA, 228 Route de L'Aérodrome, CS 40509, Domaine Saint-Paul, Site Agroparc, 84914, Avignon Cedex 9, France; b INRAE, URFM, Domaine Saint Paul, INRAE Centre de Recherche PACA, 228 Route de L'Aérodrome, CS 40509, Domaine Saint-Paul, Site Agroparc, 84914, Avignon Cedex 9, France; c INRAE, PSH, Domaine Saint Paul, INRAE Centre de Recherche PACA, 228 Route de L'Aérodrome, CS 40509, Domaine Saint-Paul, Site Agroparc, France; d Hydrosciences Montpellier, IRD, CNRS, Université de Montpellier, Montpellier, France; e Avignon Université, UMR 1114 EMMAH, 301 Rue Baruch de Spinoza, BP 21239, 84911, Avignon Cedex 9, France

**Keywords:** Xylem isotopes, Tree, ^18^O, ^2^H, Mediterranean forest, Karst

## Abstract

Water isotopes from plant xylem and surrounding environment are increasingly used in eco-hydrological studies. Carrière et al. [1] analyzed a dataset of water isotopes in (i) the xylem of three different tree species, (ii) the surrounding soil and drainage water and (iii) the underlying karst groundwater, to understand tree water uptake during drought in two different Mediterranean forests on karst setting. The xylem and soil water were extracted by cryogenic distillation. The full dataset was obtained with Isotope Ratio Mass Spectrometry (IRMS) and Isotope Ratio Infrared Spectrometer (IRIS), and included 219 measurements of δ^2^H and δ^18^O. Prompted by unexpected isotopic data characterized by a very negative deuterium excess, a subsample of 46 xylem samples and 9 soil water samples were double checked with both analytical techniques. IRMS and IRIS analyses yielded similar data. Therefore, the results reveal that laser spectrometry allows an accurate estimation of xylem and soil water isotopes. The dataset highlights a strong ^2^H depletion in xylem water for all species. Deuterium does not seem adequate to interpret ecological processes in this dataset given the strong fractionation.

**Table:** Specifications Table

Subject	Environmental Science (General)
Specific subject area	Water isotope analysis in eco-hydrology
Type of data	Table Image Graph
How data were acquired	The samples were collected in the field, on mature trees of *Quercus ilex, Fagus sylvatica* and *Abies alba* and in the soil, as part of an eco-hydrological monitoring during two summer periods with contrasting levels of drought (2014–2015). The xylem water and soil water samples were extracted by cryogenic distillation. The isotopic analyzes were made both by Isotope Ratio Mass Spectrometry (IRMS) and Isotope Ratio Infrared Spectrometer (IRIS). Liquid samples (rain and groundwater) were analyzed with laser spectrometry.
Data format	Raw
Parameters for data collection	Samples were taken during two summer periods at a monthly time step. The xylem was taken at midday from three to five trees.
Description of data collection	Each sample was composed of three to four sunny or shade twigs, collected (simply cut) from each tree at midday. Most samples were sun-exposed twigs taken from the top of the canopy (76 samples) and 13 samples of shade twigs were collected for comparison with sunny twigs. Bark and phloem were removed to prevent interference with enriched water from the leaves. The twigs were immediately packed in parafilm and placed individually in sealed vials and then transferred in a portable cooler to prevent evaporation. Soil has been collected every 10 cm through pedologic pits and samples were immediately placed in plastic bags and then transferred in a portable cooler to prevent evaporation. Liquid samples (rain, drainage water and groundwater) were directly collected within vials in smoked glass.
Data source location	- Rustrel forest above LSBB laboratory (http://lsbb.eu/presentation/): 43°56′12.15″N; 5°27′58.18″E; 530 m.a.s.l. - Mont Ventoux North Slope: 44°10′43.93″N; 5°14′36.85″E; 1340 m.a.s.l. Both sites are located within Fontaine-de-Vaucluse/LSBB observatory [2].
Data accessibility	Repository name: Tree xylem isotope analysis by mass and laser spectrometry Data identification number: 10.17632/mvfvpmnxgs.2 Direct URL to data: https://data.mendeley.com/datasets/mvfvpmnxgs/3
Related research article	Carrière, S.D., Martin-StPaul, N.K., Cakpo, C.B., Patris, N., Gillon, M., Chalikakis, K., Doussan, C., Olioso, A., Babic, M., Jouineau, A., Simioni, G., Davi, H., 2020. The role of deep vadose zone water in tree transpiration during drought periods in karst settings – Insights from isotopic tracing and leaf water potential. Science of The Total Environment 699, 134332. https://doi.org/10.1016/j.scitotenv.2019.134332

**Table:** 

**Value of the Data** • This dataset is useful for researchers interested in water isotope monitoring in plants • Mass and laser spectrometers produce similar isotopic measurements of xylem and drainage water • A large isotopic fractionation is observed between soil and xylem waters, especially on deuterium

## 1 Data

This article describes groundwater, soil, drainage, rain and xylem water isotopes sampled at different dates on two experimental sites -Mont-Ventoux and Rustrel-in the Mediterranean area of Fontaine-de-Vaucluse observatory, France [2]. Xylem and drainage water were analyzed with two distinct devices (Isotope Ratio Mass Spectrometry (IRMS) and Isotope Ratio Infrared Spectrometer (IRIS)). The dataset is presented below to highlight: (i) the consistency between the two analytical methods and (ii) the relationship between δ^18^O and δ^2^H.

**Fig. 1:**
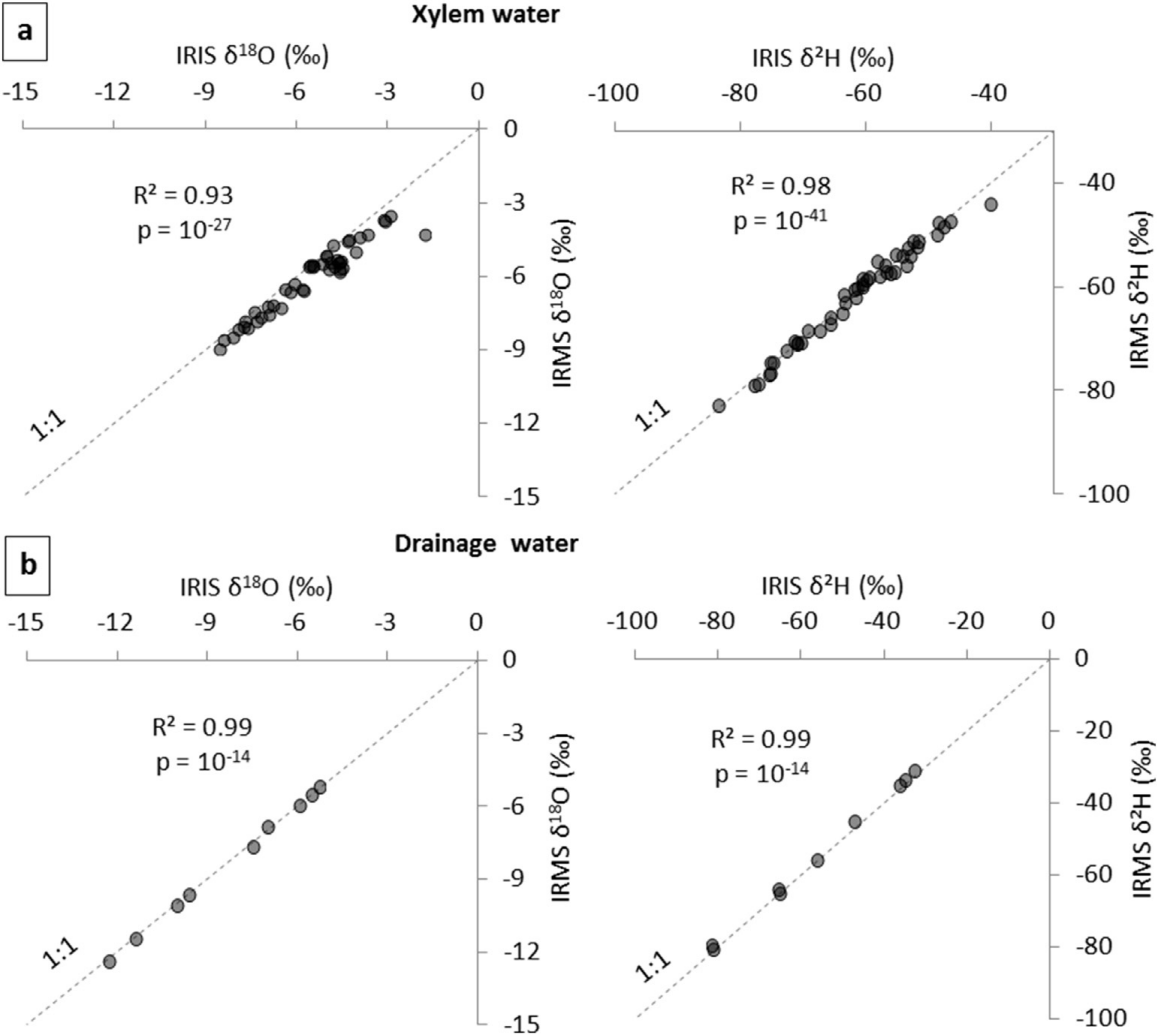
Comparison of analyzes by Isotope Ratio Mass Spectrometry (IRMS) and Isotope Ratio Infrared Spectrometer (IRIS) for: a) xylem samples and b) drainage water.

Firstly, on Fig. 1, analyzes performed by IRIS were compared to those performed by IRMS. This figure is split in two: the xylem water (Fig. 1a) and the drainage water (Fig. 1b) for both ^18^O and ^2^H isotopes. There is a strong correlation for drainage measured by IRIS and IRMS (R^2^ > 0.99; p < 0.001) while the correlation is a little weaker for xylem water analyzes (R^2^ = 0.93 to 0.98 and p < 0.001). This statement is not fully consistent with the paper of Martín-Gómez et al. [3] who showed a strong impact of organic carbon on lRIS analysis. They probably worked with higher organic carbon concentrations. Only one xylem water point is clearly shifted from the line 1:1. This point is a sample for which the fiberglass filter within the cryogenic extraction line had been forgotten. This water sample was brown colored due to a higher content in fine wood particles, which may have produced interferences in analyses. The mean difference between the two analytical methods for the 46 double checked xylem water samples (as Δ = δ_IRMS_-δ_IRIS_) are −0.45 ± 0.45‰ for δ^18^O, and −0.05 ± 1.35‰ for δ^2^H. Whereas the discrepancies may be somewhat higher than the precision reported for both methods (cf. section “Water extraction and isotopic analyzesˮ).

**Fig. 2:**
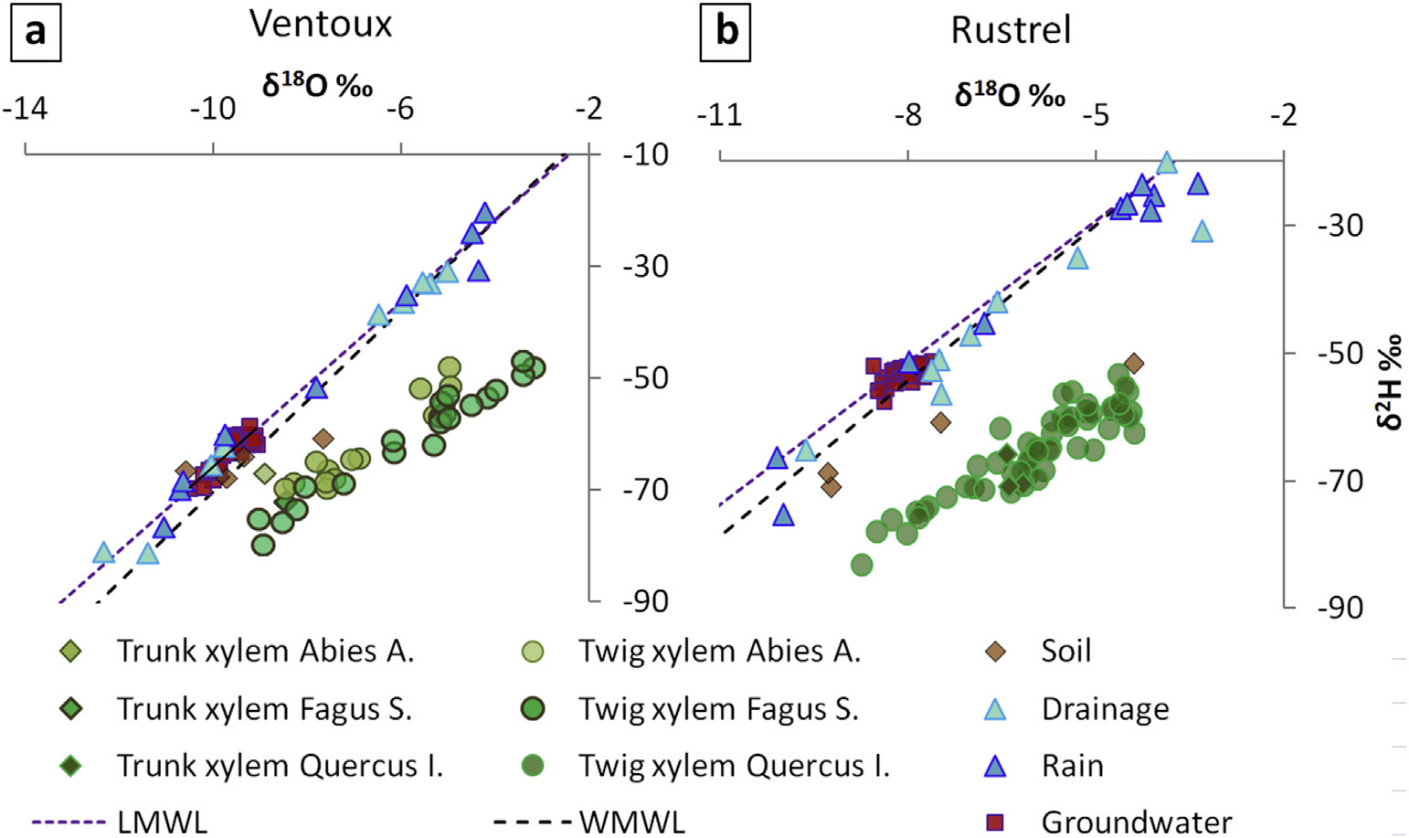
δ^18^O vs δ^2^H dataset from IRMS for the two experimental sites: a) Rustrel and b) Mont Ventoux (WMWL: world meteoric water line; LMWL: local meteoric water line [12]).

Secondly, Fig. 2 relates the ^18^O and ^2^H data through a dual isotope plot δ^18^O vs. δ^2^H. It is noticeable that rainwater, drainage and groundwater points are globally along global and local meteoric water lines. Soil water groups along a typical apparent evaporation line [4]. Evaporation is larger for the driest site (Rustrel), as expected. The xylem waters form a cloud of points clearly shifted downwards from the meteoric water line causing a very negative deuterium excess. This pattern is a result of a deuterium fractionation. Such isotopic fractionation of δ^2^H was noted in many studies [e.g. [[5], [6], [7], [8], [9], [10], [11]]].

## 2 Experimental design, materials, and methods

### 2.1 Isotopic sampling

**Fig. 3:**
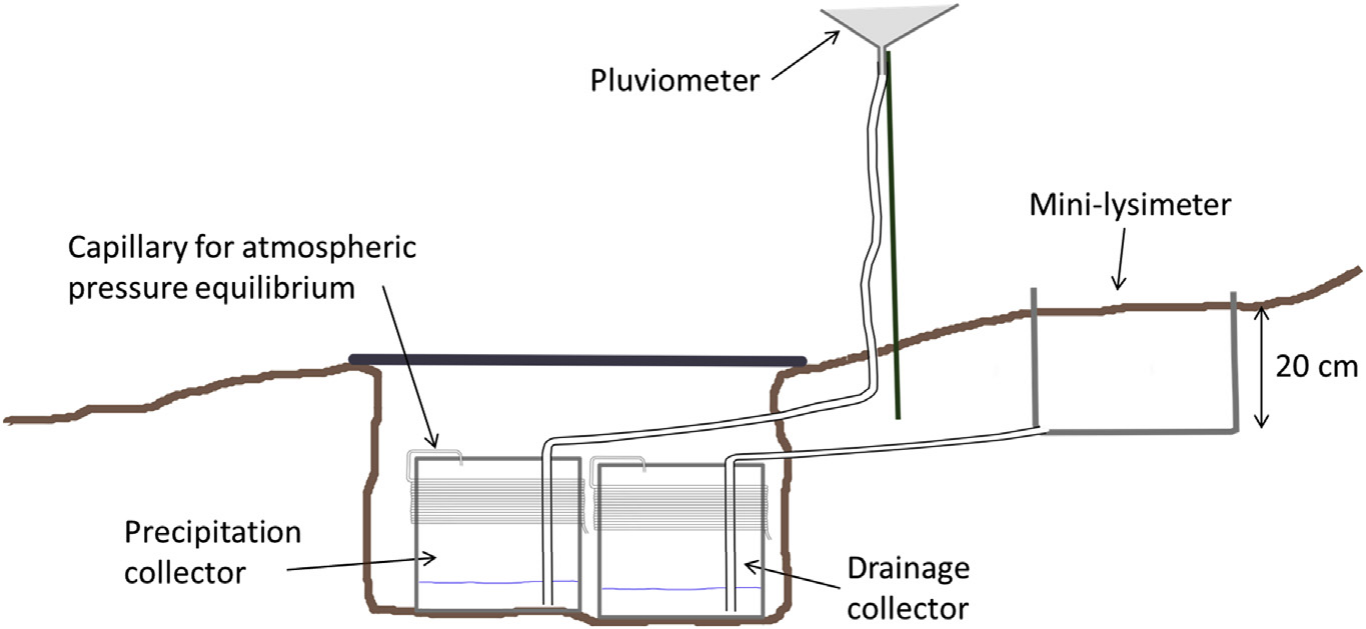
Rain and drainage water collecting system.

All the measurements were repeated at monthly time steps during two contrasted summer periods in term of drought. This dataset was designed to study the isotopic signal of trees under different levels of water stress (Carrière et al. [1]). Field work was conducted between July 2014 and August 2015. Three tree species were sampled during two successive summer periods. The xylem of holm oak (*Quercus ilex L.*), beech (*Fagus sylvatica L.*) and silver fir (*Abies alba M.*) was taken from two experimental sites. Wood twigs were sampled on three to five trees every one to two months. Three to four sun-exposed or shade twigs were collected from each tree at midday. Bark and phloem were removed to prevent interference with enriched water from the leaves. The twigs were immediately packed in parafilm and placed individually in sealed vials and then transferred in a portable cooler to prevent evaporation. All samples were stored frozen at the laboratory until water was extracted and analyzed. A corer was used to sample xylem from the trunk on one date (June 2015) at each site (Mont Ventoux and Rustrel). Samples conservation was similar to that of the twigs. Precipitation and drainage water were collected every one to three weeks between July 2014 and August 2015. Precipitation was collected using pluviometers and stored in containers buried in a pit sheltered from light to limit temperature variations (Fig. 3). In addition, these containers were equipped with an atmospheric pressure capillary in accordance to the International Atomic Energy Agency (IAEA) protocol [13] in order to limit exchanges between collected water and the atmosphere (Fig. 3). At each site, drainage water was collected at 20 cm below the surface through a mini-lysimeter of 400 cm^2^ (Fig. 3). This gravitational water was directed to a container similar to those used for precipitation (Fig. 3). We chose to collect drainage water because the subsurface is too rocky for auger drilling or ceramic porous cup sampling.

Two groundwater locations were sampled for each site: i) seepage D and C in the tunnel of Low Noise Underground Laboratory (LSBB) of Rustrel, ii) the Contrat spring (Ctr) and the Mont Serein spring (MtS) at the Mont Ventoux site.

### 2.2 Water extraction and isotopic analyzes

Water from xylem samples was extracted by cryogenic vacuum distillation [14,15]. The twigs were quickly cut into small pieces and placed in an electrothermal heating and stirring mantle at 90–100 °C for 1 hour. The water was collected in two successive liquid nitrogen traps. Generally, 3–5 ml of xylem water were extracted. These liquid samples were stored in small vials until analyzed.

Precipitation and deep-water samples were only analyzed on a Los Gatos Isotope Ratio Infrared Spectrometer (IRIS) at the University of Avignon (LGR DLT-100 liquid water stable analyzer accuracy ±0.2‰ vs V-SMOW for δ^18^O and ±1‰ vs V-SMOW for δ^2^H). In order to cancel the potential memory effect of the IRIS analyzer, each measurement was repeated 6 times and only the last 4 measurements were used to calculate the average isotope signal. However, because of possible spectral perturbations of IRIS measurements due to organic contaminants in xylem and drainage samples [3], δ^18^O and δ^2^H of xylem and drainage samples were measured using both the Los Gatos IRIS and the Isoprime IRMS at the LAMA laboratory of HydroSciences Montpellier. IRMS measurement of δ^18^O was performed using the CO_2_ equilibration technique in dual inlet mode, yielding δ^18^O analysis with a ±0.06‰ precision. IRMS measurement of δ^2^H was performed with a Eurovector PyrOH Elemental Analyzer converting H_2_O to H_2_ on hot Cr powder (1070 °C), coupled with the Isoprime in continuous-flow mode. The precision for δ^2^H was ±0.8‰. For both isotopes, the samples were compared to 3 homemade standards calibrated against IAEA reference water. The isotopic ratios, e.g. for ^18^O, are expressed as:

(1) $$\delta^{18}O=\left[\left(\frac{R_{sample}}{R_{standard}}\right)-1\right]\times 1000‰$$

where *R*_*sample*_ and *R*_*standard*_ are the heavy/light isotope ratios (^18^O/^16^O) of the sample and the standard (Vienna Standard Mean Ocean Water (VSMOW)), respectively.
